# Encryption of Color Images with an Evolutionary Framework Controlled by Chaotic Systems

**DOI:** 10.3390/e25040631

**Published:** 2023-04-07

**Authors:** Xinpeng Man, Yinglei Song

**Affiliations:** School of Automation, Jiangsu University of Science and Technology, Zhenjiang 212003, China; 202030060@stu.just.edu.cn

**Keywords:** image encryption, color images, evolutionary process, chaotic systems, security

## Abstract

In the past decade, a large amount of important digital data has been created and stored in the form of color images; the protection of such data from undesirable accesses has become an important problem in information security. In this paper, a new approach based on an evolutionary framework is proposed for the secure encryption of color images. The image contents in a color image are first fully scrambled with a sequence of bit-level operations determined by a number of integer keys. A scrambled image is then encrypted with keys generated from an evolutionary process controlled by a set of chaotic systems. Analysis and experiments show that the proposed approach can generate encrypted color images with high security. In addition, the performance of the proposed approach is compared with that of a few state-of-the-art approaches for color image encryption. The results of the comparison suggest that the proposed approach outperforms the other approaches in the overall security of encrypted images. The proposed approach is thus potentially useful for applications that require color image encryption.

## 1. Introduction

With the rapid development of information technologies, a tremendous amount of image data has been created for the analysis, storage and transmission of important information. In practice, undesirable accesses to certain image data often need to be prevented and encryption is a computational technique extensively utilized to enhance the security of such images [1]. Since images encrypted by traditional encryption methods generally cannot reach the security levels required by many applications [2], researchers have proposed numerous methods that can encrypt images with improved security [2,3,4,5,6,7]. Most of the existing approaches use techniques from one of three major classes of methods for image encryption. These classes consist of approaches that apply the permutation of pixel positions [7,8], techniques that transform gray values of pixels [5,9,10] and methods that encrypt images with chaotic systems [6,11].

An important method for image encryption permutes the positions of pixels to generate a cipher image. For example, a skew tent map system is combined with a permutation–diffusion architecture in [7] for image encryption. In [8], cipher images are generated by changing both gray values and locations of pixels. The approach proposed in [12] utilizes ergodic matrices to change the locations of pixels for image encryption. The approach proposed in [13] uses an elliptic curve random generator and an AES to improve the security of cipher images. Peano–Hilbert curves are used in [14] to relocate pixels such that spatial correlations can be eliminated in cipher images. In [15], edge maps generated based on source images are used along with a number of different permutation techniques for image encryption.

Gray value transformation is another technique that has been extensively used for image encryption. The image contents in each pixel of an image are processed by a transformation and new pixel values are generated for the pixel. The approach proposed in [9] encrypts an image multiple times with the fractional Fourier Transform (FRFT). In [5], images are iteratively encrypted by an approach that uses gyrator transform and random phase encoding. The approach proposed in [16] encrypts an image with a combination of Arnold transformation and gyrator transformation. In [8], the hue(H), saturation(S) and intensity(I) components of a color image are processed with discrete fractional random transform (DFRNT) and Arnold transform for encryption. In [17], image encryption is performed based on a new category of Discrete Fractional Fourier Transforms (DFrFs) with eigensystems generated by a new random-matrix scheme. The approach proposed in [18] uses DNA encoding to diversify the Elliptic Curve Cryptography (ECC) to obtain cipher images with improved security. A novel modular approach is proposed in [19] to construct a nonlinear S-box for image encryption. Self-adaptive permutation–diffusion and deoxyribonucleic acid (DNA) random encoding are combined in [20] for the adaptive encryption of images. The work in [21] encrypts images with an approach that uses both compressive sensing and information hiding. In [22], an approach that integrates Arnold map, DNA sequence operations with a Mandelbrot set are proposed to securely encrypt color images. Recently, DNA coding and compressive sensing have been combined to obtain cipher images with improved security [23]. The work in [24] proposes a novel layer-based image steganography method that can hide a color image into color images.

A large number of approaches have been proposed to perform image encryption with chaotic systems [3,6]. Such approaches generally utilize outputs of chaotic systems for the generation of cipher images. Since a tiny amount of change in initial values can alter the behavior of a chaotic system significantly, the exact initial values of these systems must be obtained to decipher a cipher image. The security of a cipher image can thus be significantly enhanced when chaotic systems are used for encryption. Chaotic systems are used in [4] to generate chaotic sequences that can change the locations of pixels for encryption. In [10], three sets of pseudo random sequences are generated with a simple perception and a high-dimensional chaotic system to encrypt images. In [25], images are encrypted with a pseudo-random key stream sequence generated by a piecewise linear chaotic map. In [26], an image is encrypted with two sets of one-dimensional logistic systems designed for pixel relocation and gray value transformation, respectively. The work in [27] designs a neotype chaotic product trigonometric map (PTM) system for image encryption. The approach proposed in [28] combines chaotic systems with particle swarm optimization algorithm to improve the security of color image encryption. In [29], a dual permutation and dual substitution framework that utilizes cellular automata, chaos theory and image mixing is proposed for color image encryption. The work in [30] proposes an Enhanced Logistic Map (ELM) that can be combined with chaotic maps and simple encryption techniques to improve the security of cipher images.

Recently, numerous methods that combine DNA operations with chaotic systems have been developed for image encryption [22,31,32,33]. The approach proposed in [31] encrypts images with a combination of DNA sequence operations and chaotic systems. The work in [34] performs image fusion encryption with an approach based on hyper-chaotic systems and DNA sequence operations. A hybrid model is proposed in [35] to combine DNA masking, Lorenz system and a Secure Hash Algorithm SHA-2 for image encryption. In [36], highly secure cipher images are obtained by combining DNA sequence operations with various types of chaotic systems. The work in [33] proposes a method that performs image encryption with a 5D hyper chaotic system and DNA technology.

Research results have shown that using chaotic systems together with other encryption approaches can also improve the security of cipher images. In [37], global chaotic pixel diffusion is used together with fractal sorting matrices (FSM) for image encryption. The approach proposed in [38] applies the Knuth–Durstenfeld algorithm with a hidden attractor chaos system to improve the security of cipher images. In [39], color images are encrypted with a two-dimensional logistic tent modular map (2D-LTMM). In [40], chaotic systems are integrated with a permutation–substitution (SP) network to achieve improved security for image encryption. In [41], images are encrypted by chaotic data generated with Mixed Linear–Nonlinear Coupled Map Lattices (MLNCML).

Existing methods for image encryption have significantly improved the overall security of cipher images. However, most of the state-of-the-art approaches for image encryption only change the locations of pixels to reduce the local correlations among pixels. The image contents associated with a pixel are completely or partially retained after it is relocated to a different position in the image. The original image contents are thus not completely eliminated by the scrambling process. Moreover, the transformations utilized to change the image contents in each pixel are generally based on numeric functions only. The security of cipher images can possibly be further improved if more sophisticated transformations are available for encoding the image contents associated with each pixel in an image. A new approach that can perform the scrambling process more effectively and apply a more sophisticated transformation to change the image contents associated with each pixel is thus highly desirable to achieve further improved security for image encryption.

In this paper, a new method is proposed to encrypt color images with an evolutionary framework controlled by a set of chaotic systems. In the first phase of the encryption, pixels in a color image are grouped based on a virtual image constructed from the original image and arrays formed by the binary bits in the pixel groups are shuffled with a set of integer keys. The R, G and B components of pixels are then determined from the shuffled arrays as the result of scrambling. In the second phase of encryption, an evolutionary system comprised of integers obtained from a set of 3D chaotic systems is used to change the image contents in each pixel of the scrambled image. Integers in the evolutionary system are processed and changed by two operators, including cross-over and mutation. The operations of each operator are controlled by a set of one-dimensional logistic systems. 

The proposed method has two major contributions for the encryption of color images. Firstly, a bit-level scrambling process is developed to change the location of each bit in the image contents of a color image. Correlations that usually exist between pixels that are spatially close in natural images can thus be significantly reduced. Secondly, due to the fact that it is difficult to simulate the outputs of an evolutionary process controlled by chaotic systems with numeric transformations, the underlying mechanisms of the proposed approach can be well hidden from adversary sides. The proposed method can thus significantly enhance the robustness and security of cipher images.

The results of an analysis on the key space of the proposed approach suggest that it is robust against exhaustive attacks. Experimental results on benchmark color images and a set of images selected from the BSD500 dataset [42] show that the proposed approach can generate cipher images with high security. In addition, the performance of the proposed approach is compared with that of other state-of-the-art encryption approaches on a variety of security measures for cipher images. The results of comparison show that the proposed approach is able to provide cipher images with security higher than those generated by other tested approaches.

## 2. Materials and Methods

The encryption of a color image with the proposed approach is performed in two phases. In the first phase, the binary bits of the R, G and B components in the pixels of the plain image are scrambled based on a set of integer keys and a virtual image constructed from the plain image. In the second phase, the R, G and B components in each pixel of the scrambled image are encoded by an evolutionary system constructed from the outputs of a set of 3D chaotic systems. The evolutionary process of the system is controlled by a set of one-dimensional logistic systems. 

**Figure 1:**
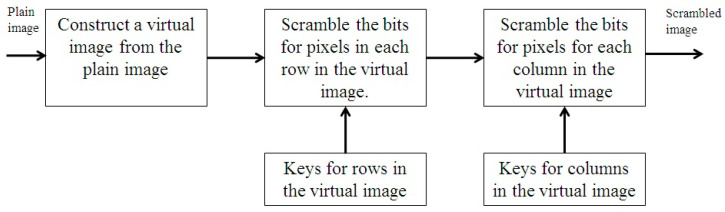
Steps in the scrambling process of the proposed approach.

Figure 1 illustrates the scrambling process in the proposed approach. In the first step, a virtual image is constructed from the plain image by a mapping that can place each pixel in the plain image to its mapped location in the virtual image. Pixels in the plain image are organized into groups based on the rows and columns of the virtual image. In the second step, the pixels in the same row are included in a group and the binary bits of the R, G and B components of the pixels in the same group are combined into an array of bits. The bits in each array are shuffled with an integer key associated with the corresponding row. The R, G and B components of pixels are replaced by bits from the corresponding locations in the shuffled arrays. In the third step, the pixels in the same column are included in a group and the same bit-shuffling operation is performed for pixels in each group. The resulting image is a scrambled image of the plain image.

**Figure 2:**
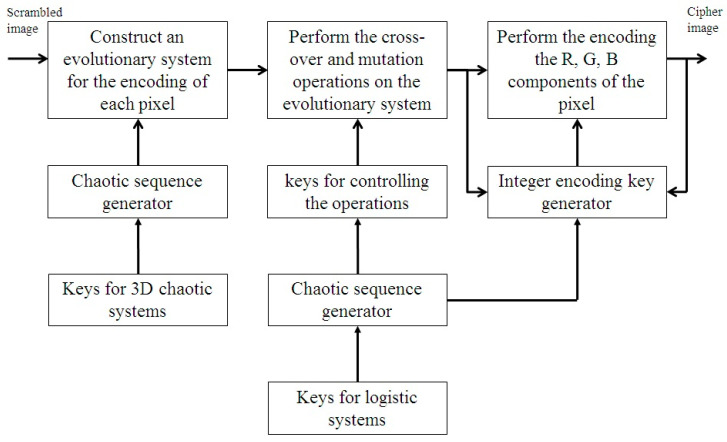
Steps in the encoding process of the proposed approach.

Figure 2 shows the steps followed by the proposed approach to encode the R, G and B components of pixels in a scrambled image. For each pixel, an evolutionary system is constructed from the outputs of a set of 3D chaotic systems. The integers in the evolutionary system are then varied by a number of cross-over and mutation operations controlled by a set of one-dimensional logistic systems. Finally, an integer is selected for each component of the pixel from the integers in the evolutionary system and the component is encoded by an integer key computed from the selected integer. 

The decryption of a cipher image can be performed in two steps. In the first step, the evolutionary system used for the encoding of each pixel is constructed and the corresponding cross-over and mutation operations are applied to the integers in the system. The integers selected for encoding the R, G and B components are obtained from the system and the components of the pixel in the scrambled image are computed from the selected integers. In the second step, the virtual image used for the scrambling process in encryption is constructed and pixels are grouped by columns in the virtual image. A reversed bit-shuffling operation is performed to reset the R, G and B components of pixels for each group. In the third step, pixels are grouped by rows in the virtual image and a reversed bit-shuffling operation is performed to reset the R, G and B components of pixels for each group. The resulting image is the plain image decrypted from the given cipher image.

### 2.1. The Bit-Level Scrambling Process 

Let I(m,n,3) be a plain color image that contains m rows and n columns; the R, G and B components of the pixel in the jth row and kth column are denoted by I(j,k,1), I(j,k,2) and $I\left(j,k,3\right)$, respectively, where 1≤j≤m and 1≤k≤n hold for integers j and k. A bit-level scrambling of I considers the binary bits in the R, G and B components of all pixels in I and changes the location of each bit in the image, the resulting image S(m,n,3) is a scrambled image of I. 

#### 2.1.1. Virtual Image

As the first step of the scrambling process, a virtual image V(p,q,3) is generated from I, where q is a given integer that satisfies $2\leq q\leq \left\lfloor mn/2\right\rfloor$ and $p=\left\lceil mn/q\right\rceil$ holds for p. V is obtained from I by a mapping Ψ that associates each pixel in I with a pixel in V. Specifically, for each pair of integers (j,k) where 1≤j≤m and 1≤k≤n hold, Ψ maps the pixel in the jth row and kth column in I to a pixel in row $R\left(j,k\right)$ and column C(j,k) in V, where R(j,k) and C(j,k) are determined based on Equations (1) and (2).

(1) $$R\left(j,k\right)=\left\{\begin{array}{ll} 1, & if\;j=1\;and\;k=1\\ \left\lceil \frac{\left(j-1\right)n+k-1}{q}\right\rceil , & otherwise \end{array}\right.$$

(2) $$C\left(j,k\right)=\left(\left(j-1\right)n+k-1\right)\;mod\;q+1$$

In addition, the following equations hold for each pair of integers (j,k) where j and k satisfy 1≤j≤m and 1≤k≤n.

(3) $$V\left(R\left(j,k\right),C\left(j,k\right),1\right)=I(j,k,1)$$

(4) $$V\left(R\left(j,k\right),C\left(j,k\right),2\right)=I(j,k,2)$$

(5) $$V\left(R\left(j,k\right),C\left(j,k\right),3\right)=I(j,k,3)$$

#### 2.1.2. Scrambling Based on Rows in the Virtual Image

It is clear from Equations (1) and (2) that row p in V may contain less than q pixels mapped from I. For each integer h that satisfies 1≤h≤p, let $r_{h}$ be the number of mapped pixels in row h of V; row h in V is assigned a positive integer $k_{h}$ for scrambling. $k_{h}$ is required to be coprime with 24q for 1≤h<p and $k_{p}$ must be coprime with $24r_{p}$.

For each integer h that satisfies 1≤h≤p, the 8-bit binary representations of the R, G and B components of the pixels in row h of V are sequentially combined into an array of $24r_{h}$ binary bits. Specifically, let $B_{h}$ be the array of binary bits constructed for row h and l be an integer that satisfies $1\leq l\leq r_{h}$; the binary bits from positions $24\left(l-1\right)+1$ through to $24\left(l-1\right)+8$ in $B_{h}$ are the 8-bit binary representation of V(h,l,1). Similarly, the binary bits from positions $24\left(l-1\right)+9$ through to $24\left(l-1\right)+16$ in $B_{h}$ are the 8-bit binary representation of V(h,l,2) and the binary bits from positions $24\left(l-1\right)+17$ through to 24l in $B_{h}$ are the 8-bit binary representation of V(h,l,3). The order of bits in $B_{h}$ is changed for each integer h that satisfies 1≤h≤p to complete the row-based scrambling operation. 

Let s be an integer that satisfies $1\leq s\leq 24r_{h}$; $B_{h}(s)$ denotes the sth bit in $B_{h}$. $B_{h}(s)$ is relocated to position t in $B_{h}$, where t is determined from $k_{h}$ and $r_{h}$ based on Equation (6).

(6) $$t=\left(\left(s-1\right)k_{h}+2k_{h}-1\right)mod\;24r_{h}+1$$

**Figure 3:**
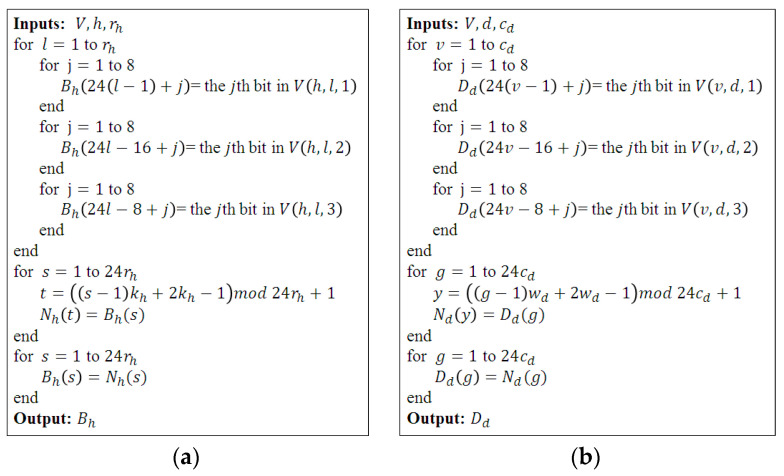
Pseudocodes for scrambling. (**a**) Scrambling the binary bits of pixels in row h in the virtual image; (**b**) scrambling the binary bits of pixels in column d in the virtual image.

Since $k_{h}$ is coprime with $24r_{h}$, no two bits in $B_{h}$ are relocated to the same position in $B_{h}$. Otherwise, there exists two different integers $s_{1}$ and $s_{2}$ that satisfy $1\leq s_{1}\leq 24r_{h}$ and $1\leq s_{2}\leq 24r_{h}$, and are relocated to the same position based on Equation (6). This implies that Equation (7) holds for $s_{1}$ and $s_{2}$.

(7) $$\left(s_{1}-s_{2}\right)k_{h}=24r_{h}u$$

where u is an integer. Since $k_{h}$ is coprime with $24r_{h}$, Equation (7) implies that $24r_{h}$ is a factor of $s_{1}-s_{2}$. However, due to the fact that $0<\left|s_{1}-s_{2}\right|<24r_{h}$ holds, $24r_{h}$ cannot be a factor of $s_{1}-s_{2}$, which is a contradiction. This implies that no such pair of integers exists and a relocation of all bits in $B_{h}$ can be performed based on Equation (6). Figure 3a shows the pseudocode for scrambling the binary bits of pixels in row h of the virtual image.

After all bits in $B_{h}$ have been relocated, the R, G and B components of pixels in row h of V are reset based on $B_{h}$. Specifically, for each integer l that satisfies $1\leq l\leq r_{h}$, the 8-bit binary representation of V(h,l,1) is reset to be the binary bits from positions $24\left(l-1\right)+1$ through to $24\left(l-1\right)+8$ in $B_{h}$. Similarly, the 8-bit binary representation of V(h,l,2) is reset to be the binary bits from positions $24\left(l-1\right)+9$ through to $24\left(l-1\right)+16$ in $B_{h},$ and the 8-bit binary representation of V(h,l,3) is reset to be the binary bits from positions $24\left(l-1\right)+17$ through to 24l in $B_{h}$.

#### 2.1.3. Scrambling Based on Columns in the Virtual Image

Similarly, Equations (4) and (5) suggest that a column in V may contain p or p−1 pixels mapped from I. For each integer d that satisfies 1≤d≤q, $c_{d}$ denotes the number of mapped pixels in column d of V. A positive integer $w_{d}$ is assigned to column d in V and $w_{d}$ must satisfy the requirement that it is coprime with $24c_{d}$. 

For each integer d such that 1≤d≤q holds, an array of $24c_{d}$ binary bits is constructed from a sequential combination of the 8-bit binary representations for the R, G and B components of the pixels in column d of V. Specifically, $D_{d}$ denotes the array of binary bits obtained from pixels in column d; for integer v that satisfies $1\leq v\leq c_{d}$, the 8-bit binary representation of V(v,d,1) is placed in positions $24\left(v-1\right)+1$ through to $24\left(v-1\right)+8$ of $D_{d}$. Similarly, the 8-bit binary representation of V(v,d,2) is placed in positions $24\left(v-1\right)+9$ through to $24\left(v-1\right)+16$ of $D_{d}$ and the 8-bit binary representation of V(v,d,3) is placed in positions $24\left(v-1\right)+17$ through to 24v of $D_{d}$. A complete column-based scrambling operation changes the location of each bit in $D_{d}$ for each integer d that satisfies 1≤d≤q.

Let g be an integer that satisfies $1\leq g\leq 24c_{d}$ and $D_{d}(g)$ be the gth bit in $D_{d}$. The location of $D_{d}(g)$ is changed to position y in $D_{d}$, where y is determined from $w_{d}$ and $c_{d}$ based on Equation (8).

(8) $$y=\left(\left(g-1\right)w_{d}+2w_{d}-1\right)mod\;24c_{d}+1$$

Based on an argument similar to the one presented in Section 2.1.2, no two bits in $D_{d}$ are relocated to the same position in $D_{d}$ due to the fact that $w_{d}$ is coprime with $24c_{d}$. Equation (8) thus generates a relocation of all bits in $D_{d}$. Figure 3b shows the pseudocode for scrambling the binary bits of pixels in column d of the virtual image.

After the locations of all bits in $D_{d}$ have been changed, $D_{d}$ is used to reset the R, G and B components of pixels in column d of V. For each integer v that satisfies $1\leq v\leq c_{d}$, the binary bits from positions $24\left(v-1\right)+1$ through to $24\left(v-1\right)+8$ in $D_{d}$ are assigned to V(v,d,1), the binary bits from positions $24\left(v-1\right)+9$ through to $24\left(v-1\right)+16$ in $D_{d}$ are assigned to V(v,d,2) and the binary bits from positions $24\left(l-1\right)+17$ through to 24l in $D_{d}$ are assigned to V(v,d,3). A scrambled image S(m,n,3) is obtained for I(m,n,3) after the column-based scrambling operation is completed. In practice, the operations in the scrambling process can be performed multiple times to further improve the security of encryption.

#### 2.1.4. Recover a Plain Image from Its Scrambled Image

Let q be the number of columns in the virtual image used to scramble the plain image in encryption. The virtual image V(p,q,3) used for the scrambling of the plain image is constructed from S, where $p=\left\lceil mn/q\right\rceil$. For each pair of integers (j,k) where 1≤j≤m and 1≤k≤n hold, the pixel that corresponds to (j,k) in V is obtained from S based on Equations (9)–(11).

(9) $$V\left(R\left(j,k\right),C\left(j,k\right),1\right)=S(j,k,1)$$

(10) $$V\left(R\left(j,k\right),C\left(j,k\right),2\right)=S(j,k,2)$$

(11) $$V\left(R\left(j,k\right),C\left(j,k\right),3\right)=S(j,k,3)$$

where R(j,k) and C(j,k) are obtained based on Equations (1) and (2).

For each integer d that satisfies 1≤d≤q, let $c_{d}$ be the number of mapped pixels in column d of V and $w_{d}$ be the integer key associated with column d in V for scrambling. A sequential combination of the 8-bit binary representations for the R, G and B components of the pixels in column d of S are performed to construct an array $D_{d}$ of $24c_{d}$ binary bits. Specifically, for each integer v that satisfies $1\leq v\leq c_{d}$, the binary bits in positions $24\left(v-1\right)+1$ through to $24\left(v-1\right)+8$ of $D_{d}$ are the 8-bit binary representation of S(v,d,1), the binary bits in positions $24\left(v-1\right)+9$ through to $24\left(v-1\right)+16$ of $D_{d}$ are the 8-bit binary representation of S(v,d,2), and the binary bits in positions $24\left(v-1\right)+17$ through to 24v of $D_{d}$ are the 8-bit binary representation of S(v,d,3).

**Figure 4:**
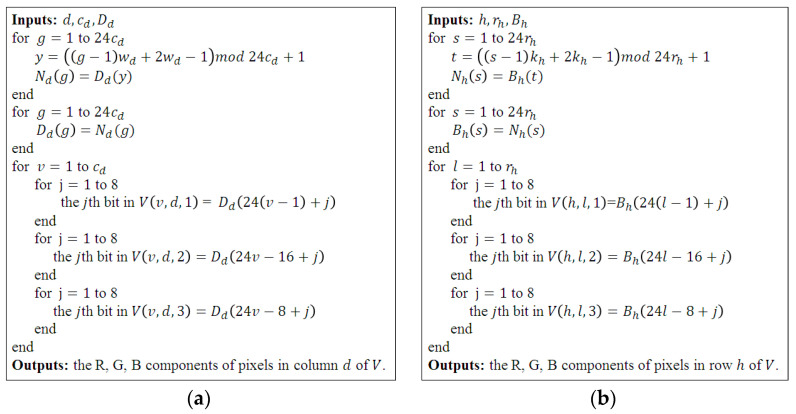
Pseudocodes for the recovery of a plain image from its scrambled image. (**a**) Recovering the binary bits of pixels in column d in the virtual image; (**b**) recovering the binary bits of pixels in row h in the virtual image.

Figure 4a shows the pseudocode for recovering the binary bits in column d of the virtual image. For each integer g such that $1\leq g\leq 24c_{d}$ holds, an integer y is determined from $w_{d}$ and $c_{d}$ using Equation (8). The location of $D_{d}(y)$ is changed to position g in $D_{d}$. After the locations of all bits in $D_{d}$ are changed, $D_{d}$ is used to reset the R, G and B components of pixels in column d of V. For each integer v that satisfies $1\leq v\leq c_{d}$, V(v,d,1) is set to be the value of the binary bits from positions $24\left(v-1\right)+1$ through to $24\left(v-1\right)+8$ in $D_{d}$, V(v,d,2) is set to be the value of the binary bits from positions $24\left(v-1\right)+9$ through to $24\left(v-1\right)+16$ in $D_{d}$ and V(v,d,3) is set to be the value of the binary bits from positions $24\left(v-1\right)+17$ through to 24v in $D_{d}$. 

For each integer h that satisfies 1≤h≤p, let $r_{h}$ be the number of mapped pixels in row h of V and $k_{h}$ be the integer key associated with row h for scrambling. The 8-bit binary representations of the R, G and B components of the pixels in row h of V are sequentially combined into an array $B_{h}$ of $24r_{h}$ binary bits. Specifically, for each integer l that satisfies $1\leq l\leq r_{h}$, the binary bits from positions $24\left(l-1\right)+1$ through to $24\left(l-1\right)+8$ in $B_{h}$ are set to be the 8-bit binary representation of V(h,l,1), the binary bits from positions $24\left(l-1\right)+9$ through to $24\left(l-1\right)+17$ in $B_{h}$ are set to be the 8-bit binary representation of V(h,l,2) and the binary bits from positions $24\left(l-1\right)+17$ through to 24l in $B_{h}$ are the 8-bit binary representation of V(h,l,3). 

Figure 4b shows the pseudocode for recovering the binary bits in row h of the virtual image. For each integer that satisfies $1\leq s\leq 24r_{h}$, an integer t is determined from $k_{h}$ and $r_{h}$ based on Equation (6), and $B_{h}(t)$ is relocated to position s in $B_{h}$. After all bits in $B_{h}$ have been relocated, the R, G and B components of pixels in row h of V are reset based on $B_{h}$. For each integer l that satisfies $1\leq l\leq r_{h}$, the value of the binary bits from positions $24\left(l-1\right)+1$ through to $24\left(l-1\right)+8$ in $B_{h}$ is assigned to V(h,l,1), the value of the binary bits from positions $24\left(l-1\right)+9$ through to $24\left(l-1\right)+16$ in $B_{h}$ is assigned to V(h,l,2) and the value of the binary bits from positions $24\left(l-1\right)+17$ through to 24l in $B_{h}$ is assigned to V(h,l,3). The plain image can be obtained after the pixels in V have been reset.

### 2.2. Encoding a Scrambled Image

#### 2.2.1. A 3D Chaotic System

Recently, a new 3D chaotic system is proposed in [43]. The system is formulated in spherical coordinates, and it is shown in [43] that four hidden attractors and three unstable equilibrium points exist for the system. The hidden attractors include two limit cycles and two strange attractors. One of the strange attractors is inside a sphere of radius 7.0 and the other one is outside the sphere. A description of the system in spherical coordinates is shown in Equation (12).

(12) $$\left\{\begin{array}{l} \dot{\rho }=\rho \varphi -7\varphi \\ \dot{\theta }=-\beta \theta^{2}+\varphi^{2}\\ \dot{\varphi }=-\rho^{2}+\alpha \theta^{2}+14\rho +\varphi -49 \end{array}\right.$$

where ρ,φ and θ are the radial distance, polar angle and azimuthal angle, respectively. The simulation results show that the system demonstrates a chaotic behavior when parameters α and β are set to be 3.0 and 1.0, respectively. A more detailed analysis of the dynamical properties of the system can be found in [43].

#### 2.2.2. The Evolutionary System for Encryption

The encryption of a scrambled image is based on an evolutionary system constructed based on a set of 3D chaotic systems described by Equation (9) with different initial values. Let b be a positive integer and $I_{1}=\{\rho_{1,0},\rho_{2,0},\ldots ,\rho_{b,0}\}$, $I_{2}=\{\theta_{1,0},\theta_{2,0},\ldots ,\theta_{b,0}\}$ and $I_{3}=\left\{\varphi_{1,0},\varphi_{2,0},\ldots \varphi_{b,0}\right\}$ be three number sets that contain the initial values for b different 3D chaotic systems $K_{1},K_{2},\ldots ,K_{b}$. Specifically, for integer i such that 1≤i≤b holds, $\rho_{i,0}$, $\theta_{i,0}$ and $\varphi_{i,0}$ are the initial values of ρ, θ and φ for system $K_{i}$. For the pixel in row j and column k in S(m,n,3), let $L=\left(j-1\right)n+k-1$; three sets of integers $E_{1,0}=\{X_{1,L,}X_{2,L},\ldots ,X_{b,L}\}$, $E_{2,0}=\{Y_{1,L},Y_{2,L},\ldots ,Y_{b,L}\}$ and $E_{3,0}=\{Z_{1,L},Z_{2,L},\ldots ,Z_{b,L}\}$ can be obtained with Equation (13).

(13) $$\left\{\begin{array}{c} X_{i,L}=\left\lfloor M\times {|x}_{i,L}|\right\rfloor \;mod\;256\\ Y_{i,L}=\left\lfloor M\times |y_{i,L}|\right\rfloor \;mod\;256\\ Z_{i,L}=\left\lfloor M\times |z_{i,L}|\right\rfloor \;mod\;256 \end{array}\right.$$

where i is an integer that satisfies 1≤i≤b and M is a large positive integer;$x_{i,L}$, $y_{i,L}$ and $z_{i,L}$ are computed based on Equation (14).

(14) $$\left\{\begin{array}{l} x_{i,L}=\rho_{i,L}cos\theta_{i,L}sin\varphi_{i,L}\\ y_{i,L}=\rho_{i,L}sin\theta_{i,L}sin\varphi_{i,L}\\ z_{i,L}=\rho_{i,L}cos\varphi_{i,L} \end{array}\right.$$

where $\rho_{i,L}$, $\theta_{i,L}$ and $\varphi_{i,L}$ are the outputs of $K_{i}$ at time L∆t, given $\rho_{i,0}$, $\theta_{i,0}$ and $\varphi_{i,0}$ as its initial values for ρ, θ and φ, respectively.∆t is a positive constant. 

The integer sets $E_{1,0}$, $E_{2,0}$ and $E_{3,0}$ together form the initial configuration of the evolutionary system for encoding the pixel in row j and column k in S(m,n,3). The integers in $E_{1,0}$, $E_{2,0}$ and $E_{3,0}$ are changed by a series of cross-over and mutation operations controlled by a set of one-dimensional logistic systems; the resulting sets are denoted by $E_{1,f}$, $E_{2,f}$ and $E_{3,f}$. The integer keys for the encoding of S(j,k,1), S(j,k,2) and $S\left(j,k,3\right)$ are computed based on integers selected from $E_{1,f}$, $E_{2,f}$ and $E_{3,f}$, respectively.

#### 2.2.3. The Cross-Over Operation

A cross-over operation for the evolutionary system is controlled by a set of one-dimensional logistic systems. A logistic system is defined by an initial value $l_{0}$ and the recursion relation shown in Equation (15).

(15) $$l_{i+1}=4l_{i}\left(1-l_{i}\right)$$

where i is a positive integer. A well-known fact is that the logistic system defined in Equation (15) is a one-dimensional chaotic system when $l_{0}$ satisfies $0<l_{0}<1$ [44]. The system defined in Equation (15) generates a sequence of numbers $l_{1},l_{2},\ldots ,l_{N},\ldots$ for a given initial value $l_{0}$. Due to the chaotic property of the system, a tiny amount of change in $l_{0}$ would lead to a significantly different $l_{N}$ if N is a large enough integer.

Three one-dimensional logistic systems with different initial values are needed to complete a cross-over operation. The initial values of the three one-dimensional logistic systems are denoted by $l_{1,0}$, $l_{2,0}$ and $l_{3,0}$. For the pixel in row j and column k in S(m,n,3), three sets of integers $V_{1,0}=\{u_{1,L,}u_{2,L},\ldots ,u_{b,L}\}$, $V_{2,0}=\{v_{1,L},v_{2,L},\ldots ,v_{b,L}\}$ and $V_{3,0}=\{z_{1,L},z_{2,L},\ldots ,z_{b,L}\}$ can be obtained based on Equation (16).

(16) $$\left\{\begin{array}{c} u_{i,L}=\left\lfloor M_{0}\times l_{1,Lb+i-1}\right\rfloor \;mod\;7+1\\ v_{i,L}=\left\lfloor M_{0}\times l_{2,Lb+i-1}\right\rfloor \;mod\;7+1\\ z_{i,L}=\left\lfloor M_{0}\times l_{3,Lb+i-1}\right\rfloor \;mod\;7+1 \end{array}\right.$$

where $M_{0}$ is a large positive integer and $l_{1,Lb+i-1}$, $l_{2,Lb+i-1}$ and $l_{3,Lb+i-1}$ are the Lb+i−1th elements in the chaotic number sequences generated from Equation (15) with $l_{1,0}$, $l_{2,0}$ and $l_{3,0}$ as the initial values, respectively.

The cross-over operation is performed based on $V_{1,0}$, $V_{2,0}$ and $V_{3,0}$. In addition, three positive integer keys $g_{1}$, $g_{2}$ and $g_{3}$ are needed to determine the pairs of integers where the cross-over operations need to be performed in $V_{1,0}$, $V_{2,0}$ and $V_{3,0}$, respectively. $g_{1}$,$g_{2}$ and $g_{3}$ are all coprime with b. 

To perform the cross-over operation on $E_{1,0}$, for each integer e that satisfies 1≤e≤b, $X_{e,L}$ is paired with $X_{a_{1},L}$ for cross-over, where $a_{1}$ is determined by Equation (17).

(17) $$a_{1}=\left(\left(e-1\right)g1+2g_{1}-1\right)\;mod\;b+1$$

Based on $u_{e,L}$, two new integers $X_{e,L}^{\prime }$ and $X_{a,L}^{\prime }$ are generated by Equations (18) and (19).

(18) $$X_{e,L}^{\prime }=\left\lfloor X_{e,L}/2^{u_{e,L}}\right\rfloor +X_{a_{1},L}\;mod\;2^{u_{e,L}}$$

(19) $$X_{a,L}^{\prime }=\left\lfloor X_{a_{1},L}/2^{u_{e,L}}\right\rfloor +X_{e,L}\;mod\;2^{u_{e,L}}$$

As the result of the cross-over operation, $X_{e,L}$ is replaced by $X_{e,L}^{\prime }$ and $X_{a_{1},L}$ is replaced by $X_{a_{1},L}^{\prime }$.

The cross-over operation on $E_{2,0}$ is performed with a similar method. For each integer e that satisfies 1≤e≤b, a pair between $Y_{e,L}$ and $Y_{a_{2},L}$ is formed for cross-over, and $a_{2}$ is obtained from Equation (20).

(20) $$a_{2}=\left(\left(e-1\right)g_{2}+2g_{2}-1\right)\;mod\;b+1$$

Two new integers $Y_{e,L}^{\prime }$ and $Y_{a_{2},L}^{\prime }$ are obtained from $v_{e,L}$ by Equations (21) and (22).

(21) $$Y_{e,L}^{\prime }=\left\lfloor Y_{e,L}/2^{v_{e,L}}\right\rfloor +Y_{a_{2},L}\;mod\;2^{v_{e,L}}$$

(22) $$Y_{a_{2},L}^{\prime }=\left\lfloor Y_{a_{2},L}/2^{v_{e,L}}\right\rfloor +Y_{e,L}\;mod\;2^{v_{e,L}}$$

The cross-over operation replaces $Y_{e,L}$ by $Y_{e,L}^{\prime }$ and $Y_{a_{2},L}$ is replaced by $Y_{a_{2},L}^{\prime }$. 

Similarly, the cross-over operation on $E_{3,0}$ is performed on integer pairs formed based on $g_{3}$. For each integer e such that 1≤e≤b holds, an integer pair is generated between $Z_{e,L}$ and $Z_{a_{3},L}$ for cross-over, and $a_{3}$ is computed based on Equation (23).

(23) $$a_{3}=\left(\left(e-1\right)g_{3}+2g_{3}-1\right)\;mod\;b+1$$

Two new integers $Z_{e,L}^{\prime }$ and $Z_{a_{3},L}^{\prime }$ are determined from $z_{e,L}$ by Equations (24) and (25).

(24) $$Z_{e,L}^{\prime }=\left\lfloor Z_{e,L}/2^{z_{e,L}}\right\rfloor +Z_{a_{3},L}\;mod\;2^{z_{e,L}}$$

(25) $$Z_{a_{3},L}^{\prime }=\left\lfloor Z_{a_{3},L}/2^{z_{e,L}}\right\rfloor +Z_{e,L}\;mod\;2^{z_{e,L}}$$

**Figure 5:**
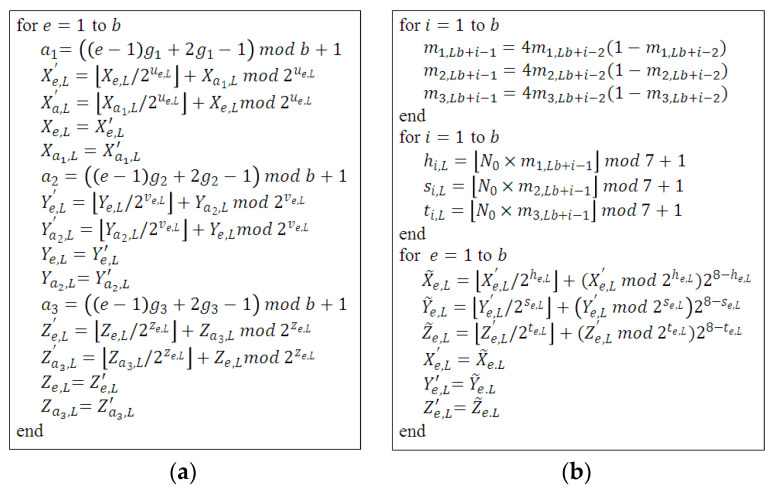
Pseudocodes for the cross-over and mutation operations. (**a**) Cross-over operation; (**b**) mutation operation.

The cross-over operation substitutes $Z_{e,L}$ and $Z_{a_{3},L}$ with $Z_{e,L}^{\prime }$ and $Z_{a_{3},L}^{\prime }$, respectively. Figure 5a shows the pseudocode for a cross-over operation.

#### 2.2.4. The Mutation Operation

Let $E_{1,c}=\{X_{1,L}^{\prime }$,$X_{2,L}^{\prime },\ldots ,X_{b,L}^{\prime }\}$, $E_{2,c}=\{Y_{1,L}^{\prime },Y_{2,L}^{\prime },\ldots ,Y_{b,L}^{\prime }\}$ and $E_{3,c}=\{Z_{1,L}^{\prime },Z_{2,L}^{\prime },\ldots ,Z_{b,L}^{\prime }\}$ be the integer sets generated from $E_{1,0}$, $E_{2,0}$ and $E_{3,0}$ by a cross-over operation. A mutation operation performs a rotational right shift on each integer in $E_{1,c}$, $E_{2,c}$ and $E_{3,c}$. The number of bit positions by which the shift is performed for an integer is determined by a set of three one-dimensional logistic systems. Let $m_{1,0}$, $m_{2,0}$ and $m_{3,0}$ be the initial values of the three logistic systems; three sets of integers $M_{1,0}=\{h_{1,L,}h_{2,L},\ldots ,h_{b,L}\}$,$M_{2,0}=\{s_{1,L},s_{2,L},\ldots ,s_{b,L}\}$ and $M_{3,0}=\{t_{1,L},t_{2,L},\ldots ,t_{b,L}\}$ are generated based on Equation (26) for the pixel in row j and column k in S(m,n,3).

(26) $$\left\{\begin{array}{c} h_{i,L}=\left\lfloor N_{0}\times m_{1,Lb+i-1}\right\rfloor \;mod\;7+1\\ s_{i,L}=\left\lfloor N_{0}\times m_{2,Lb+i-1}\right\rfloor \;mod\;7+1\\ t_{i,L}=\left\lfloor N_{0}\times m_{3,Lb+i-1}\right\rfloor \;mod\;7+1 \end{array}\right.$$

where $N_{0}$ is a large positive integer and $m_{1,Lb+i-1}$, $m_{2,Lb+i-1}$ and $m_{3,Lb+i-1}$ are the Lb+i−1th elements in the chaotic number sequences obtained from Equation (15) using $m_{1,0}$, $m_{2,0}$ and $m_{3,0}$ as the initial values, respectively. 

Integers in $M_{1,0}$, $M_{2,0}$ and $M_{3,0}$ provide the number of bit positions by which each integer in $E_{1,c}$, $E_{2,c}$ and $E_{3,c}$ needs to be rotationally shifted to complete the mutation operation. For each integer e such that 1≤e≤b holds, $X_{e,L}^{\prime }$, $Y_{e,L}^{\prime }$ and $Z_{e,L}^{\prime }$ are rotationally shifted to the right by $h_{e,L}$, $s_{e,L}$ and $t_{e,L}$ bit positions, respectively. In other words, $X_{e,L}^{\prime }$, $Y_{e,L}^{\prime }$ and $Z_{e,L}^{\prime }$ are replaced by ${\tilde{X}}_{e,L}$, ${\tilde{Y}}_{e,L}$ and ${\tilde{Z}}_{e,L}$ generated by Equations (27), (28) and (29), respectively. The resulting integer sets in the evolutionary system are denoted by $E_{1,f}$, $E_{2,f}$ and $E_{3,f}$. Figure 5b shows the pseudocode for a mutation operation.

(27) $${\tilde{X}}_{e,L}=\left\lfloor X_{e,L}^{\prime }/2^{h_{e,L}}\right\rfloor +{(X}_{e,L}^{\prime }\;mod\;2^{h_{e,L}})2^{8-h_{e,L}}$$

(28) $${\tilde{Y}}_{e,L}=\left\lfloor Y_{e,L}^{\prime }/2^{s_{e,L}}\right\rfloor +\left(Y_{e,L}^{\prime }\;mod\;2^{s_{e,L}}\right)2^{8-s_{e,L}}$$

(29) $${\tilde{Z}}_{e,L}=\left\lfloor Z_{e,L}^{\prime }/2^{t_{e,L}}\right\rfloor +{(Z}_{e,L}^{\prime }\;mod\;2^{t_{e,L}})2^{8-t_{e,L}}$$

#### 2.2.5. Encoding of Pixels

An integer is selected from each of the three integer sets $E_{1,f}=\{{\tilde{X}}_{1,L},{\tilde{X}}_{2,L},\ldots ,{\tilde{X}}_{b,L}\}$, $E_{2,f}=\{{\tilde{Y}}_{1,L},{\tilde{Y}}_{2,L},\ldots ,{\tilde{Y}}_{b,L}\}$ and $E_{3,f}=\{{\tilde{Z}}_{1,L},{\tilde{Z}}_{2,L},\ldots ,{\tilde{Z}}_{b,L}\}$ to generate integer keys that can encode the pixel in row j and column k in S(m,n,3). The selection is controlled by a one-dimensional logistic system with an initial value of $s_{0}$; three integers $c_{1,L}$, $c_{2,L}$ and $c_{3,L}$ are obtained based on Equation (30) for the pixel in row j and column k in S(m,n,3).

(30) $$\left\{\begin{array}{l} c_{1,L}=\left\lfloor N_{1}\times s_{3L}\right\rfloor \;mod\;b+1\\ c_{2,L}=\left\lfloor N_{1}\times s_{3L+1}\right\rfloor \;mod\;b+1\\ c_{3,L}=\left\lfloor N_{1}\times s_{3L+2}\right\rfloor \;mod\;b+1 \end{array}\right.$$

where $N_{1}$ is a large positive integer and $s_{3L}$, $s_{3L+1}$ and $s_{3L+2}$ are the 3lth, 3l+1th and 3l+2th elements, respectively, in the chaotic number sequences obtained from Equation (15) using an initial value of $s_{0}$.

Let C(m,n,3) be the cipher image obtained from S(m,n,3); the integer keys used to encode S(j,k,1), S(j,k,2) and S(j,k,3) are denoted by $I_{k}(j,k,1)$, $I_{k}(j,k,2)$ and $I_{k}(j,k,3)$, respectively. $I_{k}(j,k,1)$, $I_{k}(j,k,2)$ and $I_{k}(j,k,3)$ are obtained based on Equation (31).

(31) $$\left\{\begin{array}{c} I_{k}\left(j,k,1\right)=\left(H_{j,k}{\tilde{X}}_{c_{1,L},L}\right)\;mod\;256\\ I_{k}\left(j,k,2\right)=\left(H_{j,k}{\tilde{Y}}_{c_{2,L},L}\right)\;mod\;256\\ I_{k}\left(j,k,3\right)=\left(H_{j,k}{\tilde{X}}_{c_{3,L},L}\right)\;mod\;256 \end{array}\right.$$

where $H_{j,k}$ is 1 when j=1 and k=1; otherwise,$H_{j,k}=\sum_{v=1}^{3}C(j_{p},k_{p},v)$, where $j_{p}$ and $k_{p}$ can be computed from j and k with Equations (32) and (33).

(32) $$j_{p}=\left\{\begin{array}{ll} 1, & if\;j=1\;and\;k=2\\ \left\lceil \frac{\left(j-1\right)n+k-2}{n}\right\rceil , & otherwise \end{array}\right.$$

(33) $$k_{p}=\left(\left(j-1\right)n+k-2\right)\;mod\;q+1$$

Finally, C(i,j,1), C(i,j,2) and C(i,j,3) are determined from Equation (34).

(34) $$\left\{\begin{array}{c} C\left(j,k,1\right)=S(j,k,1)⊕I_{k}(j,k,1)\\ C\left(j,k,2\right)=S(j,k,2)⊕I_{k}(j,k,2)\\ C\left(j,k,2\right)=S(j,k,3)⊕I_{k}(j,k,3) \end{array}\right.$$

Equations (31)–(33) show that the encryption keys $I_{k}(j,k,1)$, $I_{k}(j,k,2)$ and $I_{k}(j,k,3)$ are dependent on the encrypted image contents associated with the pixel in row $j_{p}$ and column $k_{p}$. This fact suggests that the proposed approach is plain-image-sensitive. In practice, the security of encryption can be further enhanced by applying the encoding process multiple times.

#### 2.2.6. Decoding a Cipher Image

The scrambled image of a cipher image can be obtained based on the initial values of the 3D chaotic systems used to construct the evolutionary system for encoding and the initial values of the one-dimensional logistic systems that control the evolutionary system during the encoding.

Let C(m,h,3) be a cipher image.$I_{1}=\{\rho_{1,0},\rho_{2,0},\ldots ,\rho_{b,0}\}$, $I_{2}=\{\theta_{1,0},\theta_{2,0},\ldots ,\theta_{b,0}\}$ and $I_{3}=\{\varphi_{1,0},\varphi_{2,0},\ldots \varphi_{b,0}\}$ are the sets of initial values for the b3D chaotic systems;$l_{1,0}$, $l_{2,0}$ and $l_{3,0}$ are the initial values of the logistic systems for cross-over operations;$m_{1,0}$, $m_{2,0}$ and $m_{3,0}$ are the initial values of the logistic systems for mutation operations; and $s_{0}$ is the initial value of the logistic system that selects integers from the evolutionary system for computing the encoding keys. 

To decode the pixel in row j and column k in C(m,h,3), Equations (13) and (14) are used to obtain three integer sets $E_{1,0}=\{X_{1,L,}X_{2,L},\ldots ,X_{b,L}\}$, $E_{2,0}=\{Y_{1,L},Y_{2,L},\ldots ,Y_{b,L}\}$ and $E_{3,0}=\{Z_{1,L},Z_{2,L},\ldots ,Z_{b,L}\}$ that together constitute the evolutionary system. A cross-over operation is then applied to $E_{1,0}$, $E_{2,0}$ and $E_{3,0}$ as described in Section 2.2.3 to obtain three integer sets $E_{1,c}=\{X_{1,L}^{\prime }{,X}_{2,L}^{\prime },\ldots ,X_{b,L}^{\prime }\}$, $E_{2,c}=\{Y_{1,L}^{\prime },Y_{2,L}^{\prime },\ldots ,Y_{b,L}^{\prime }\}$ and $E_{3,c}=\{Z_{1,L}^{\prime },Z_{2,L}^{\prime },\ldots ,Z_{b,L}^{\prime }\}$. The operations described in Section 2.2.4 are applied to $E_{1,c}$, $E_{2,c}$ and $E_{3,c}$ to complete the mutation operation and three integer sets $E_{1,f}=\{{\tilde{X}}_{1,L},{\tilde{X}}_{2,L},\ldots ,{\tilde{X}}_{b,L}\}$, $E_{2,f}=\{{\tilde{Y}}_{1,L},{\tilde{Y}}_{2,L},\ldots ,{\tilde{Y}}_{b,L}\}$ and $E_{3,f}=\{{\tilde{Z}}_{1,L},{\tilde{Z}}_{2,L},\ldots ,{\tilde{Z}}_{b,L}\}$ are obtained as the result. Based on $E_{1,f}$, $E_{2,f}$ and $E_{3,f}$, Equations (30)–(33) are utilized to generate $I_{k}(j,k,1)$, $I_{k}(j,k,2)$ and $I_{k}(j,k,3)$, which are the encoding keys for the pixel. S(i,j,1), S(i,j,2) and S(i,j,3) are obtained based on Equation (35).

(35) $$\left\{\begin{array}{c} S\left(j,k,1\right)=C(j,k,1)⊕I_{k}(j,k,1)\\ S\left(j,k,2\right)=C(j,k,2)⊕I_{k}(j,k,2)\\ S\left(j,k,2\right)=C(j,k,3)⊕I_{k}(j,k,3) \end{array}\right.$$

### 2.3. Computational Complexity

For a plain color image that contains m rows and n columns, a virtual image for scrambling can be constructed in O(mn) time. The bit-level scrambling based on rows and columns of a virtual image can be accomplished in O(mn) time. The scrambling process of the proposed approach thus needs O(mn) time.

The encoding process encodes each pixel in a scrambled image with an evolutionary system that contains 3b integers. A cross-over operation can be performed in O(b) time and the computation time needed for a mutation operation is O(b). The encoding of a scrambled image thus requires O(bmn) time. Therefore, the computation time needed for the encryption of an image is O(bmn). 

In practice, it is often desirable to perform the scrambling and encoding processes multiple times. For an encryption process that scrambles a plain image for $n_{s}$ times and encodes the scrambled image for $n_{e}$ times, a total amount of $O(\left(n_{s}+n_{e}b\right)mn)$ computation time is needed to complete the encryption.

## 3. Results

A computer program has been created to implement the proposed approach in MATLAB and its encryption security has been analyzed based on the cipher images generated for seven benchmark images and 100 color images selected from the BSD500 dataset [42]. In addition, a comparison of the proposed approach with several state-of-the-art encryption methods is performed based on a number of security measures. Other approaches tested for comparison are the methods proposed in [7,20,28,38,43,44,45,46,47,48,49]. In the testing, an evolutionary system that contains five 3D chaotic systems is constructed to encode pixels in a scrambled image. A value of $10^{12}$ is chosen for integers M, $M_{0}$, $N_{0}$ and $N_{1}$.

### 3.1. Brutal Force Attacks

The robustness of an encryption method against brutal force attacks can be evaluated based on the size of its key space. A larger key space usually implies stronger robustness against brutal force attacks. In the scrambling process of the proposed approach, each row or column in a virtual image is associated with an integer key for bit-level scrambling. Let $Z_{1}$ be the size of the integer set where a key for a row or column can be selected, for a virtual image with p rows and q columns, $Z_{1}^{p+q}$ different combinations exist for the integer keys utilized in scrambling process. In the encoding process, $Z_{2}^{3d+7}$ different combinations exist for the initial values of d 3D chaotic systems and seven one-dimensional logistic systems; $Z_{2}$ is the size of the set of real numbers where the initial value for a chaotic system can be selected.

The key space of the proposed approach is thus $Z_{1}^{p+q}Z_{2}^{3d+7}$. For a plain color image that contains at least $10^{4}$ pixels, the value of p+q is at least $2\sqrt{pq}\geq 200$. When five 3D chaotic systems are used for the encryption of such an image, the key space size is at least $Z_{1}^{200}Z_{2}^{22}$. In practice, both $Z_{1}>2^{10}$ and $Z_{2}>2^{60}$ hold, and the key space size for a cipher image of the image is thus at least $2^{3320}$. 

Table 1 shows the key space sizes of a few encryption methods, including the proposed approach and several other state-of-the-art approaches. It is evident from Table 1 that the proposed approach has a key space size larger than those of the other encryption methods and its robustness against brutal force attacks is thus higher than that of the other methods.

### 3.2. Statistical Attacks

**Figure 6:**
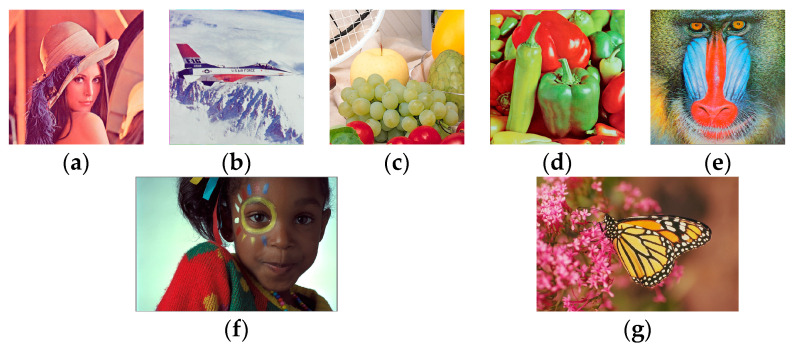
Benchmark images used for testing. (**a**) Lena; (**b**) Airplane; (**c**) Fruits; (**d**) Peppers; (**e**) Baboon; (**f**) Girl; (**g**) Monarch.

The proposed approach is applied to seven popular benchmark images for encryption and an analysis is performed on the obtained cipher images to evaluate the overall strength of the approach against potential statistical attacks. Five of the seven benchmark images, including Lena, Airplane, Fruits, Peppers and Baboon, have a size of 512 × 512 while the other two benchmark images, including Monarch and Girl, have a size of 768 × 512. The seven testing benchmark images are shown in Figure 6a–g.

#### 3.2.1. Analysis of Histograms

**Figure 7:**
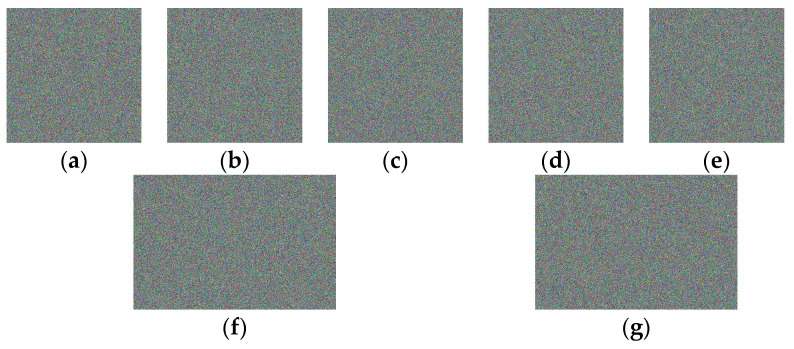
Cipher images obtained for benchmark images. (**a**) For Lena; (**b**) for Airplane; (**c**) for Fruits; (**d**) for Peppers; (**e**) for Baboon; (**f**) for Girl; (**g**) for Monarch.

The cipher image obtained by the proposed approach for each benchmark image is shown in Figure 7a–g. It is clear from Figure 7 that the image contents in a plain image are completely removed from its cipher image and all cipher images appear to be highly random. 

**Figure 8:**
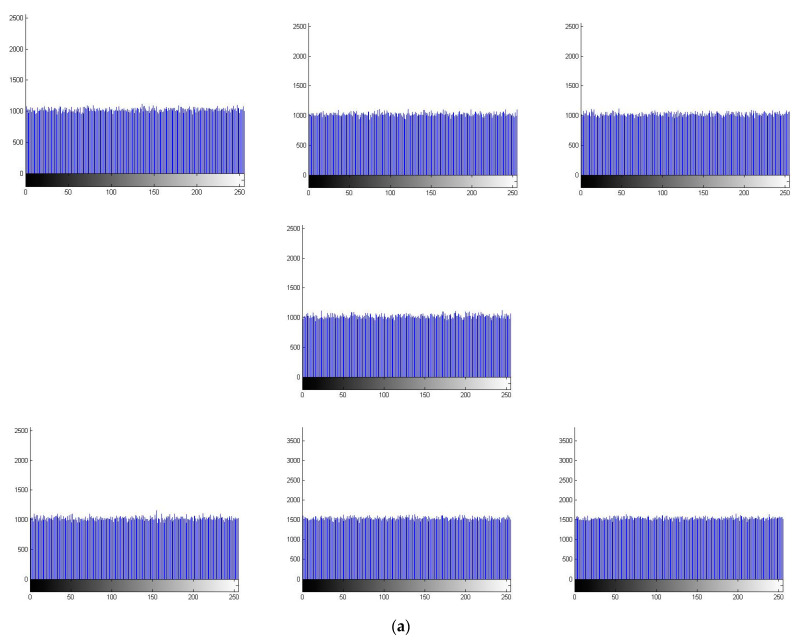

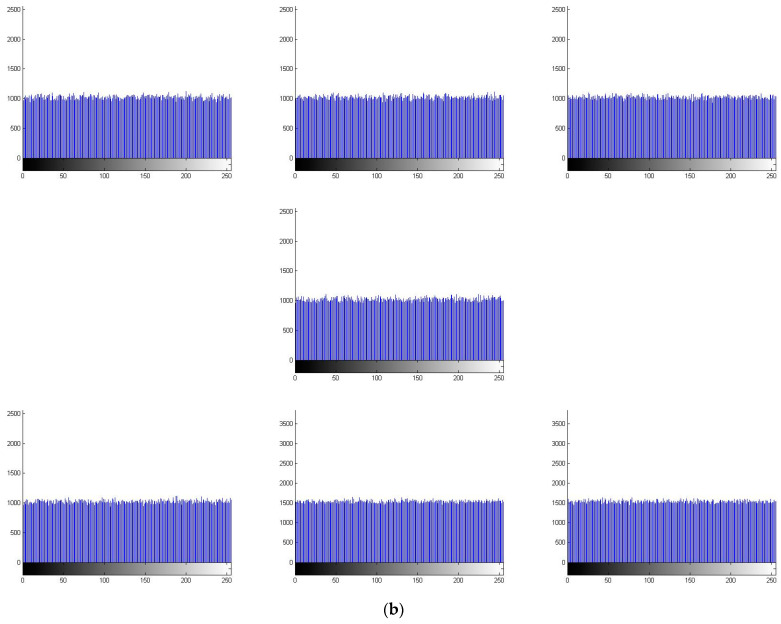

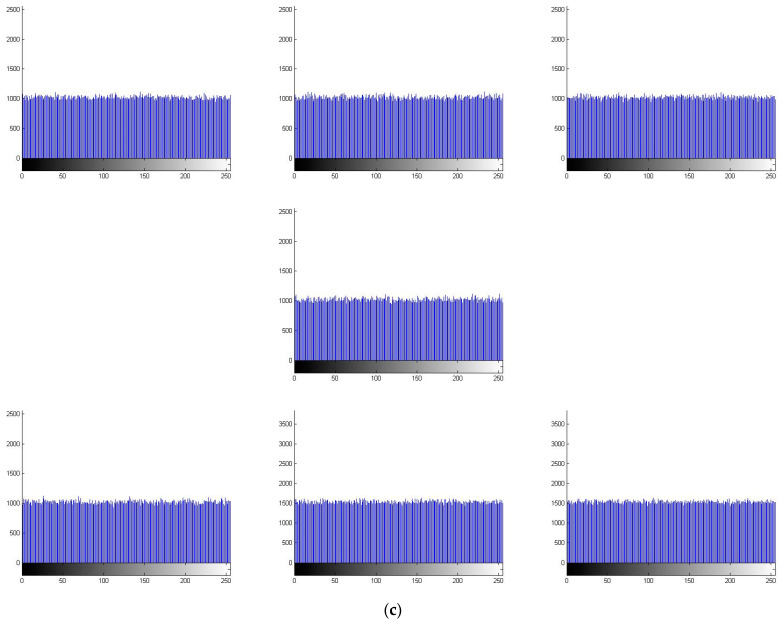
Histograms of the R, G and B components in the cipher images. From **upper left** to **lower right**: Lena, Airplane, Fruits, Peppers, Baboon, Girl and Monarch; (**a**–**c**) show the histograms of the R, G and B components, respectively.

The histograms of the R, G and B components in the cipher images of the benchmark images are shown in Figure 8a–c. It can be seen from Figure 8 that the R, G and B components in cipher images all follow near-uniform distributions and the histograms of a cipher image do not contain information related to the image contents in its plain image.

An important measure often used to evaluate the uniformity of a histogram is the variance of histograms [33]. Histogram P contains a counting value for each integer between 0 and 255. The variance of histogram Var(P) of P is calculated by Equation (36).

(36) $$Var\left(P\right)=\frac{1}{2n^{2}}\sum_{i=1}^{n}\sum_{j=1}^{n}{\left(p_{i}-p_{j}\right)}^{2}$$

where n=256 is the number of counting values in P. $p_{i}$ and $p_{j}$ are the counting values associated with integers i and j in P. Equation (36) clearly shows that histograms with lower values of variances of histograms generally are closer to a uniform distribution.

The variances of histograms for the cipher images are calculated and compared with those obtained with several other encryption methods, including methods proposed in [22,33,48,49]. The results of the comparison are shown in Table 2. Table 2 clearly shows that the proposed approach achieves the lowest value for variances of histograms on Airplane, Fruits and Monarch and it ranks the second position on Lena and Baboon. The results in Table 2 suggest that the overall performance of the proposed approach on the variances of histograms is better than that of the other methods.

#### 3.2.2. Analysis of Correlations

**Figure 9:**
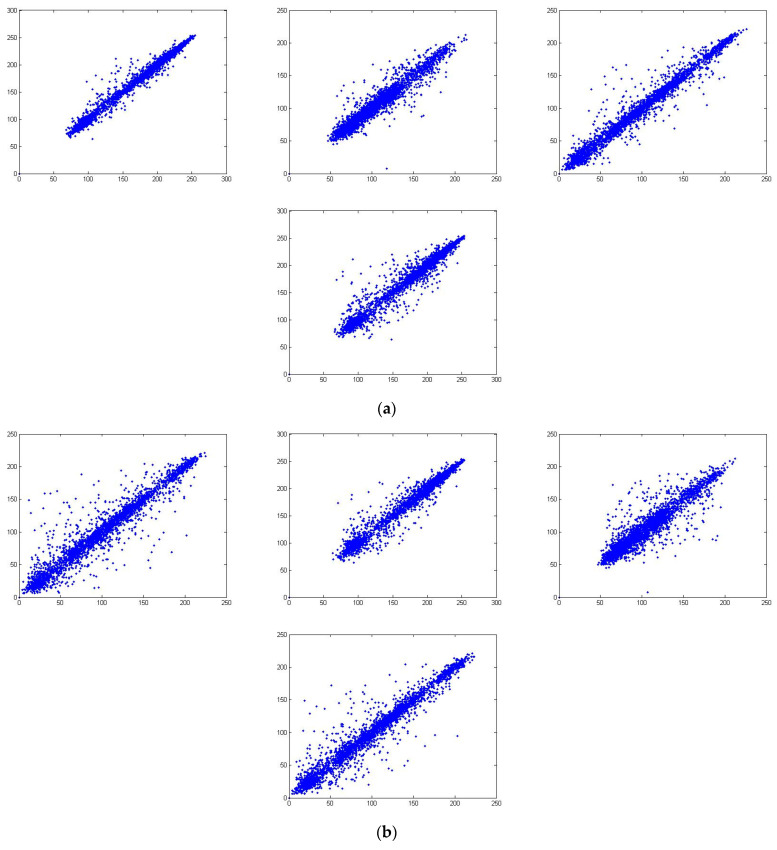

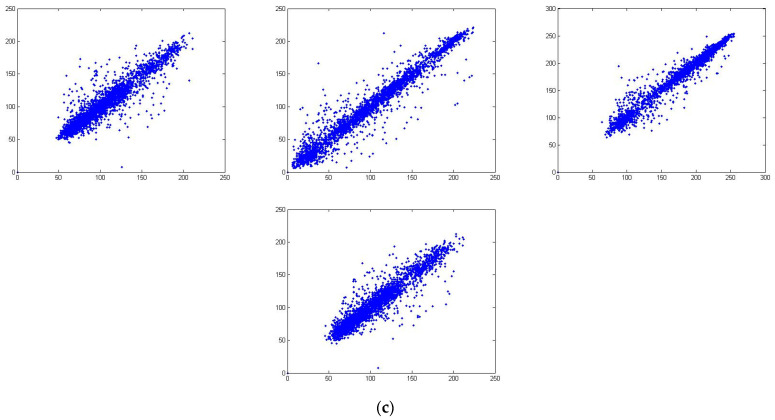
Correlations in the R, G and B components in Lena. From **left** to **right**: horizontal, vertical, diagonal and anti-diagonal; (**a**–**c**) show the correlations in the R, G and B components, respectively.

**Figure 10:**
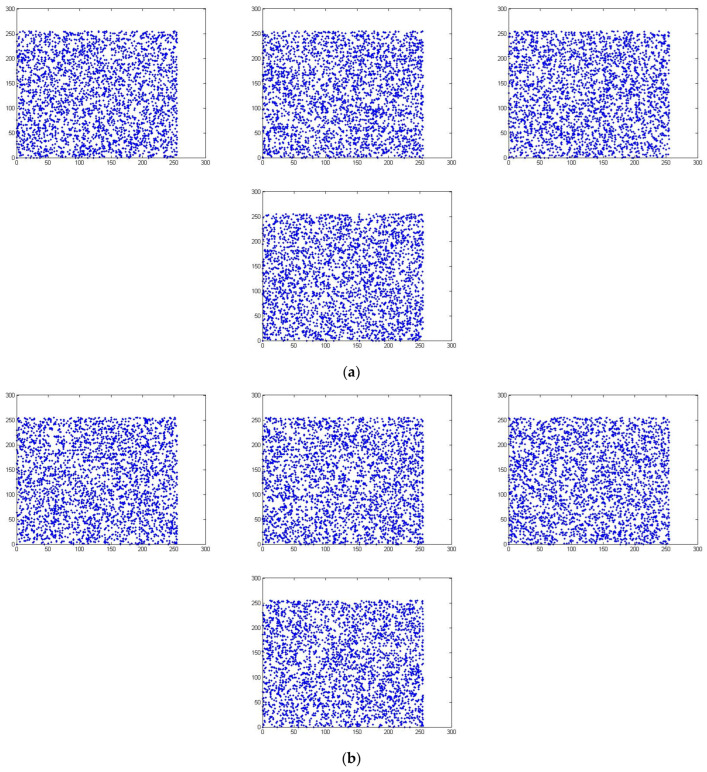

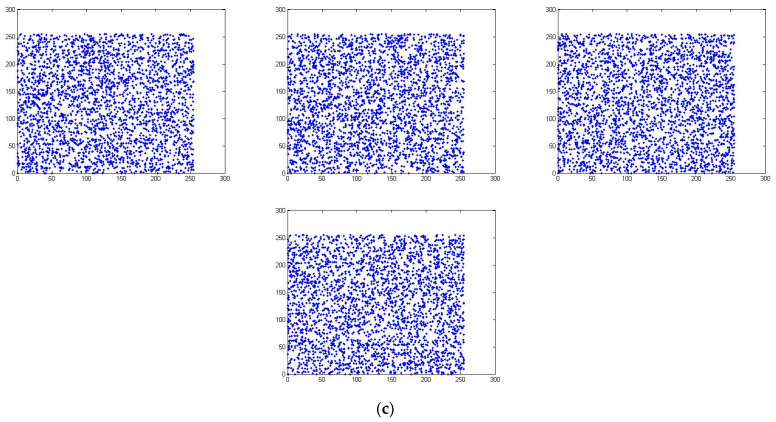
Correlations in different directions for all components in the cipher image of Lena. From **left** to **right**: horizontal, vertical, diagonal and anti-diagonal; plots of correlations in R, G and B components are shown in (**a**–**c**), respectively.

A well-known fact is that strong correlations usually exist between pixels that are spatially close in a plain color image. Such correlations are often closely associated with the contents contained in a plain image and thus need to be reduced to values close to zero when encryption is complete. In general, the correlations among pixels that are adjacent in horizontal, vertical, diagonal and anti-diagonal directions in a cipher image are used as important measures on its strength over potential statistical attacks. The correlations between adjacent pixels in the above four directions in Lena and its cipher image are plotted based on 3000 pixels randomly chosen from the images. Figure 9a–c show the plots obtained for the R, G and B components of pixels in Lena. Figure 10a–c show the plots obtained on its cipher image.

The correlations for pixels adjacent in the four directions have been obtained for all benchmark images and their cipher images; these are shown in Table 3. Table 3 suggests that the correlations between pixels adjacent in a cipher image reach values close to zero. This fact ensures that cipher images generated with the proposed approach cannot be deciphered by statistical attacks. The correlations for the R components of pixels adjacent in cipher images obtained with several different methods on Lena are compared in Table 4. The results in Table 4 suggest that the proposed approach can achieve a performance comparable with other state-of-the-art encryption methods on eliminating local correlations in cipher images.

### 3.3. Differential Attacks

The strength of an encryption approach over differential attacks is generally evaluated by its sensitivities to tiny changes in encryption keys and plain image. The Number of Pixels Change Rate (NPCR) and unified average changing intensity (UACI) are two measures often used to evaluate such sensitivities [22,35,40,52]. 

Given a plain image I with m rows and n columns, a set of keys for encryption and the resulting cipher image $C_{e_{1}}$,$C_{e_{2}}$ is the cipher image generated after one of the keys in the key set, or one of the R, G and B components in one pixel in I is changed by a tiny amount. The NPCR and UACI for component t are calculated by Equations (37) and (38), respectively.

(37) $$N\left(t\right)=\frac{\sum_{i=1}^{m}\sum_{j=1}^{n}X(i,j,t)}{mn}$$

(38) $$U\left(t\right)=\frac{\sum_{i=1}^{m}\sum_{j=1}^{n}\left|C_{e_{1}}\left(i,j,t\right)-C_{e_{2}}\left(i,j,t\right)\right|}{255mn}$$

where $X\left(i,j,t\right)$ is an integer that depends on a comparison between $C_{e_{1}}\left(i,j,t\right)$ and $C_{e_{2}}(i,j,t)$, its value is 1 if $C_{e_{1}}\left(i,j,t\right)$ and $C_{e_{2}}(i,j,t)$ are different and is 0 otherwise. In an ideal case, NPCR has a value of 99.6094 and UACI has a value of 33.4635.

To evaluate the key sensitivity of the proposed approach, a perturbation of $10^{-16}$ is applied to the initial values of the chaotic systems used for the encoding process; the values of NPCR and UACI for the R, Gand B components are calculated with Equations (36) and (37) for each testing benchmark image. Table 5 shows the values of key sensitivity NPCR and UACI obtained for each testing benchmark image. It can be seen from Table 5 that, for all components, the values of key sensitivity NPCR and UACI obtained on all testing images are close to their ideal values. 

The results in Table 5 suggest that the mean key sensitivity NPCR of the proposed approach is larger than 99.6100 and its mean key sensitivity UACI is close to 33.4571. In [52], values of 99.5893, 99.5810 and 99.5717 are established for NPCR randomness tests at levels 0.05, 0.01 and 0.001, respectively; the proposed approach thus passes the NPCR randomness tests for all three levels. Similarly, in [52], lower bound values 33.3730, 33.3445 and 33.3115 are established along with upper bound values 33.5541, 33.5826 and 33.6156 for UACI randomness tests at levels 0.05, 0.01 and 0.001, respectively. Since the mean UACI of the proposed approach is close to 33.4571, it also passes the UACI randomness tests for all three levels.

Table 6 shows the values of key sensitivity NPCR and UACI obtained with the proposed approach and several other encryption methods on Lena. It is evident from Table 6 that the mean value of key sensitivity NPCR of the proposed approach is 99.6133 on Lena and its mean value of key sensitivity UACI is 33.4600. Since the ideal values for NPCR and UACI are 99.6064 and 33.4635, respectively, the proposed approach has the best overall performance on key sensitivities. 

To evaluate the plain image sensitivity of the proposed approach, a pixel is randomly selected from a plain image and one of its R, G and B components is changed by 1. The cipher image of the resulting image is then compared with that of the original image; the NPCR and UACI for each component can then be calculated based on Equations (36) and (37).

The NPCR and UACI values for plain image sensitivity of the proposed approach on the testing images are shown in Table 7. The proposed approach can achieve a mean NPCR of 99.6147 and a mean UACI of 33.4457. Due to the fact that values of 99.5893, 99.5810 and 99.5717 are associated with levels 0.05, 0.01 and 0.001, respectively, for NPCR randomness tests [52], the proposed approach is able to pass the NPCR randomness tests for all three levels. In addition, since the UACI randomness tests at levels 0.05, 0.01 and 0.001 adopt values 33.3730, 33.3445 and 33.3115 for lower bounds and values 33.5541, 33.5826 and 33.6156 for upper bounds, respectively [52], the proposed approach passes the random tests at all levels for UACI. Table 8 compares the plain image sensitivity values obtained with the proposed approach and those of several other encryption methods on Lena. Table 8 clearly suggests that the proposed approach achieves a mean value of NPCR 99.6200 on Lena for plain image sensitivity and its mean value of plain image sensitivity UACI is 33.4567. The overall performance of the proposed approach is thus the best of all tested methods on plain image sensitivities. 

### 3.4. Analysis of Information Entropy

Information entropy is a measure often utilized to represent the randomness of a cipher image. Specifically, let C(m,n,3) be a cipher image. Equation (39) is used to calculate the information entropy associated with the tth component of C(m,n,3).

(39) $$E\left(C,t\right)=-\sum_{i=0}^{255}d\left(i,C,t\right)\log_{2}d(i,C,t)$$

where d(i,C,t) is the probability that the tth component of a pixel is i. Since a uniform distribution has an entropy of 8.0, the information entropy for each component in an ideally encrypted image is 8.0. 

The information entropies of the R, G and B components in cipher images generated with the proposed approach are shown in Table 9. It can be seen from Table 9 that the information entropies for cipher images obtained by the proposed approach are all nearly ideal.

The information entropies of the cipher images generated with the proposed approach and several other encryption methods on Lena are shown in Table 10. Table 10 clearly suggests that the proposed approach achieves the highest entropies for components R and B and ranks the second position on component G. Its overall performance on Lena is thus the same as that of the approach proposed in [37] and better than that of the other tested methods.

### 3.5. Analysis of Peak Noise Signal Ratio

The Peak Signal Noise Ratio (PSNR) provides a measure for the difference between a cipher image and its plain image. A higher PSNR value thus often suggests higher security for a cipher image [22]. The PSNR for a cipher image C(m,n,3) is calculated based on its plain color image I(m,n,3) with Equations (40) and (41).

(40) $$MSE\left(I,C\right)=\sum_{i=1}^{m}\sum_{j=1}^{n}\sum_{t=1}^{3}{\left|I\left(i,j,t\right)-C\left(i,j,t\right)\right|}^{2}$$

(41) $$PSNR\left(I,C\right)=20\log_{10}\frac{255\sqrt{3mn}}{\sqrt{MSE(I,C)}}$$

The PSNR values of the cipher images generated by the proposed approach and several other encryption approaches on testing images are shown in Table 11. It is clear from Table 11 that the overall performance of the proposed approach on PSNR is comparable to that of the method in [28] and is higher than that of the other tested methods.

### 3.6. Experimental Results on General Color Images

In addition to benchmark color images, the proposed approach is applied to encrypt 100 color images selected from the BSD500 dataset [42]. All tested images are of size 481 × 321. Its overall performance on the encryption of these images is compared with that of the methods proposed in [22,28,33,38]. Table 12 shows the means and standard deviations of the cipher images generated by the proposed approach and several other encryption methods. It is clear from Table 12 that the proposed approach outperforms the methods proposed in [22,33,38] on the variance of histograms. However, Table 12 suggests that the method in [28] is slightly better than the proposed approach on variances of histograms. Since the method in [28] optimizes the uniformity of the histograms of all components in cipher images with particle swarm optimization [53], it is not surprising that it can obtain cipher images with higher uniformity for histograms.

Table 13 compares the entropies of the cipher images obtained with the proposed approach and the other tested methods. Table 13 suggests that the proposed approach achieves the highest mean entropies for cipher images. Information on the PSNRs of the cipher images obtained with all tested methods is shown in Table 14. It can be seen from Table 14 that the proposed approach slightly outperforms all other methods in PSNR.

A comparison of the key sensitivities of the proposed approach with those of other tested methods is shown in Table 15. It is clear from Table 15 that the proposed approach can achieve mean values of NPCR and UACI closest to their ideal values. Table 16 compares the plain image sensitivities of all tested methods. The mean values of NPCR and UACI in Table 16 suggest that the overall plain image sensitivities of the proposed approach are slightly higher than those of the other tested methods.

The computational efficiency of each tested method is evaluated based on the computation time needed by the method to encrypt a color image. Table 17 shows information on the amount of computation time each method requires to generate a cipher image. Table 17 clearly shows that the computational efficiency of the proposed approach is comparable with that of the other methods.

## 4. Discussion

Although the proposed approach has been tested on a few benchmark images and general color images, additional experimental results are needed to evaluate its overall performance on general color images. Moreover, the virtual image in the scrambling process is constructed based on a pixel-level mapping, which can partially reorder the pixels in the image before the bit-level scrambling operations are performed. However, virtual images constructed based on bit-level mappings can separate bits in the same pixel well apart before the scrambling starts; scrambled images generated based on such virtual images thus may contain less information on the contents of their plain images. In addition, more sophisticated operations can possibly be developed for the evolutionary system to further improve the security of cipher images.

It is clear from Section 2 that the proposed approach may require a large number of keys to generate a cipher image. The encryption of images with large sizes thus could be computationally inefficient. In addition, the randomness of the chaotic systems used in the proposed approach may need to be further improved to enhance the security of cipher images.

A multi-dimension discrete chaotic map with excellent ergodicity and randomness is proposed in [54] for image encryption. The proposed approach can probably be combined with the chaotic map proposed in [54] for further improvement in security. Moreover, continuous chaotic systems proposed in [55] can be used with parameter perturbation to enhance randomness.

The proposed approach utilizes a set of randomly selected fixed keys for encryption and the round keys are obtained with modular operations. The method proposed in [56] can possibly be utilized to eliminate the potential weaknesses in the key expansion method used in the proposed approach.

## 5. Conclusions

In this paper, a new approach is proposed for the encryption of color images. The approach performs encryption in two phases. In the first phase, binary bits in the R, G and B components of pixels in a plain color image are scrambled based on the rows and columns of a virtual image constructed from the plain image. In the second phase, an evolutionary system controlled by a set of chaotic systems is utilized to generate the integer keys needed to encode the R, G and B components of pixels in a scrambled image. An analysis on the size of key space and testing results suggest that cipher images generated by the proposed approach are secure against various types of attacks. In addition, comparisons of the proposed approach with several other state-of-the-art approaches on a variety of security measures show that its overall performance is better than that of the other tested encryption methods.

## Figures and Tables

**Table 1:** The key space sizes of the proposed approach and several other encryption methods.

Methods	Size of Key Space
The proposed	$2^{3320}$
Ref. [28]	$2^{2092}$
Ref. [20]	$2^{199}$
Ref. [38]	$2^{170}$
Ref. [50]	$2^{138}$
Ref. [44]	$2^{241}$
Ref. [7]	$2^{104}$
Ref. [45]	$2^{128}$
Ref. [46]	$2^{128}$
Ref. [47]	$2^{128}$

**Table 2:** The variance of histograms for cipher images obtained with the proposed approach and several other encryption methods.

Images	The Proposed	Ref. [22]	Ref. [33]	Ref. [48]	Ref. [49]
Lena	1040.39	1047.40	1054.78	1043.21	1027.59
Airplane	1082.83	1132.27	1268.02	1141.38	1103.16
Fruits	951.94	1026.51	1034.23	1013.32	1082.34
Peppers	1146.23	1046.25	1037.78	1105.72	946.67
Baboon	1024.29	1098.64	901.22	1061.04	1058.13
Girl	1536.50	1513.42	1543.23	1620.12	1530.21
Monarch	1409.30	1564.39	1607.51	1653.27	1563.72

**Table 3:** The correlations for the R, Gand B components of pixels adjacent in four directions in the testing benchmark images and their cipher images obtained with the proposed approach.

Images	Directions	Plain Image			Cipher Image		
		R	G	B	R	G	B
Lena	V	0.9753	0.9666	0.9334	0.0019	−0.0013	−0.0006
	H	0.9853	0.9802	0.9558	0.0028	−0.0001	0.0022
	D	0.9734	0.9630	0.9264	−0.0011	0.0024	−0.0010
	A	0.9648	0.9536	0.9198	0.0002	0.0010	−0.0030
Airplane	V	0.9726	0.9578	0.9640	−0.0006	0.0017	−0.0001
	H	0.9568	0.9678	0.9353	0.0016	0.0011	−0.0013
	D	0.9343	0.9326	0.9146	−0.0002	0.0013	0.0034
	A	0.9350	0.9300	0.9075	−0.0013	0.0030	0.0020
Fruits	V	0.9936	0.9855	0.9265	−0.0007	−0.0025	−0.0030
	H	0.9928	0.9848	0.9192	−0.0022	0.0006	−0.0004
	D	0.9897	0.9783	0.8809	−0.0027	0.0013	−0.0020
	A	0.9868	0.9694	0.8531	0.0024	−0.0005	−0.0005
Peppers	V	0.9635	0.9811	0.9665	−0.0003	0.0023	0.0006
	H	0.9663	0.9817	0.9664	−0.0004	−0.0009	0.0021
	D	0.9563	0.9686	0.9477	−0.0009	0.0016	0.0022
	A	0.9585	0.9708	0.9477	0.0020	−0.0009	0.0034
Baboon	V	0.9235	0.8668	0.9067	0.0021	0.0006	0.0012
	H	0.8740	0.7759	0.8844	0.0011	−0.0002	−0.0029
	D	0.8649	0.7432	0.8544	0.0024	−0.0034	−0.0023
	A	0.8670	0.7494	0.8540	−0.0016	0.0037	0.0004
Girl	V	0.9811	0.9887	0.9866	−0.0019	−0.0012	−0.0004
	H	0.9901	0.9937	0.9927	0.0013	0.0014	−0.0002
	D	0.9750	0.9848	0.9823	0.0017	−0.0037	0.0024
	A	0.9772	0.9858	0.9832	−0.0019	0.0021	0.0035
Monarch	V	0.9648	0.9523	0.9566	1.7 × 10^−5^	−4.5 × 10^−5^	−0.0018
	H	0.9597	0.9453	0.9506	0.0020	−0.0018	0.0009
	D	0.9450	0.9252	0.9309	0.0016	0.0031	0.0014
	A	0.9245	0.8984	0.9118	0.0003	−8.7 × 10^−6^	−0.0036

**Table 4:** The correlations for pixels adjacent in horizontal (H), vertical (V) and diagonal (D) directions in the cipher images of Lena obtained with the proposed approach and several other encryption methods. The best values are shown in bold.

Approaches	H	V	D
The proposed	0.0028	0.0019	−0.0011
Ref. [28]	0.0023	−0.0020	0.0013
Ref. [38]	0.0046	−0.0028	0.0014
Ref. [51]	0.0027	**−0.0013**	0.0039
Ref. [33]	**0.0012**	0.0035	0.0056
Ref. [20]	−0.0026	−0.0038	0.0017
Ref. [52]	−0.0030	0.0025	**−0.0001**
Ref. [22]	0.0021	0.0018	−0.0026

**Table 5:** The values of NPCR and UACI in percentage for the key sensitivities of the proposed approach on the R, G and B components of cipher images. The best values are shown in bold.

Images	NPCR (%)			UACI (%)		
	R	G	B	R	G	B
Lena	**99.61**	99.60	99.63	33.45	33.42	33.51
Airplane	**99.61**	99.62	**99.61**	33.38	**33.47**	**33.46**
Fruits	99.62	99.62	99.60	33.38	33.49	33.45
Peppers	**99.61**	99.62	**99.61**	33.48	33.50	33.40
Baboon	**99.61**	**99.61**	99.63	33.55	33.38	33.49
Girl	**99.61**	**99.61**	**99.61**	**33.47**	33.45	33.42
Monarch	99.60	**99.61**	**99.61**	33.50	33.49	**33.46**

**Table 6:** The values of NPCR and UACI in percentage for the key sensitivities of the proposed approach and several other encryption methods on the R, G and B components of the cipher images of Lena. The best values are shown in bold.

Methods	NPCR (%)			UACI (%)		
	R	G	B	R	G	B
The proposed	**99.61**	99.60	99.63	33.45	33.42	33.51
Ref. [29]	99.62	99.62	99.62	33.48	33.45	33.50
Ref. [40]	**99.61**	**99.61**	**99.61**	**33.47**	**33.47**	**33.47**
Ref. [22]	99.57	99.58	99.57	33.35	33.37	33.38
Ref. [35]	99.60	99.59	99.61	33.45	33.45	33.45

**Table 7:** The values of NPCR and UACI in percentage for the plain image sensitivities of the proposed approach on the R, G and B components of cipher images. The best values are shown in bold.

Images	NPCR (%)			UACI (%)		
	R	G	B	R	G	B
Lena	99.62	99.62	99.62	33.47	33.43	**33.47**
Airplane	99.63	99.62	99.60	**33.46**	33.44	33.45
Fruits	**99.61**	**99.61**	**99.61**	33.45	33.44	33.48
Peppers	99.62	99.63	**99.61**	**33.46**	33.42	33.41
Baboon	**99.61**	**99.61**	99.63	33.39	33.43	**33.47**
Girl	99.60	99.62	**99.61**	33.42	33.39	33.51
Monarch	99.62	99.60	**99.61**	33.47	**33.45**	33.45

**Table 8:** The values of NPCR and UACI in percentage for the plain image sensitivities of the proposed approach and several other encryption methods on the R, G and B components of the cipher images of Lena. The best values are shown in bold.

Methods	NPCR (%)			UACI (%)		
	R	G	B	R	G	B
The proposed	99.62	99.62	99.62	**33.47**	33.43	**33.47**
Ref. [29]	**99.61**	99.63	**99.61**	33.45	**33.46**	33.45
Ref. [40]	99.60	99.58	99.59	33.44	33.43	33.43
Ref. [22]	99.58	99.57	99.58	33.34	33.34	33.34
Ref. [35]	99.59	**99.60**	99.59	33.33	33.33	33.33

**Table 9:** The information entropy for the R, G and B components of pixels in the cipher images of testing images obtained with the proposed approach. The best values are shown in bold.

Images	R	G	B
Lena	7.9993	7.9992	7.9994
Airplane	7.9993	7.9992	7.9992
Fruits	7.9993	7.9994	7.9994
Peppers	7.9992	7.9992	7.9992
Baboon	7.9992	7.9994	7.9993
Girl	7.9995	**7.9996**	7.9995
Monarch	**7.9996**	**7.9996**	**7.9996**

**Table 10:** The information entropy for the R, G and B components of pixels in the cipher images of Lena obtained with the proposed approach and several other encryption methods. The best values are shown in bold.

Methods	R	G	B
The proposed	**7.9993**	7.9992	**7.9994**
Ref. [28]	7.9992	7.9992	7.9992
Ref. [37]	7.9992	**7.9993**	**7.9994**
Ref. [22]	7.9992	7.9992	7.9992
Ref. [33]	7.9976	7.9976	7.9976

**Table 11:** The values of PSNR for the proposed approach and several other encryption methods on all testing images. The best values are shown in bold.

Images	The Proposed	Ref. [28]	Ref. [22]	Ref. [33]	Ref. [48]	Ref. [49]
Lena	**83.022**	83.021	81.131	80.927	81.027	81.225
Airplane	**83.022**	**83.022**	82.462	82.234	82.478	82.531
Fruits	83.022	**83.023**	81.023	80.835	81.147	80.632
Peppers	83.022	83.021	82.971	83.073	82.735	**83.105**
Baboon	83.022	**83.023**	82.873	83.145	82.652	82.534
Girl	**84.783**	84.782	84.653	84.641	84.437	84.685
Monarch	84.783	**84.784**	84.732	84.697	84.625	84.746

**Table 12:** The means and standard deviations of the variances of histograms for cipher images generated by the proposed approach and several other encryption methods. The best values are shown in bold.

Components		The Proposed	Ref. [28]	Ref. [38]	Ref. [22]	Ref. [33]
R	Mean	594.79	**583.19**	648.91	637.28	625.53
	STD	53.73	44.21	47.52	46.76	45.94
G	Mean	601.27	**586.32**	644.93	646.37	631.13
	STD	49.06	50.37	55.28	52.94	54.06
B	Mean	617.94	**607.25**	666.21	663.71	656.22
	STD	45.06	89.61	98.91	96.92	95.66

**Table 13:** The means and standard deviations of the information entropies for the R, G and B components of the cipher images obtained with the proposed approach and other tested encryption methods. The best values are shown in bold.

Methods	R		G		B	
	Mean	STD	Mean	STD	Mean	STD
The proposed	**7.9988**	0.0001	**7.9988**	0.0001	**7.9988**	0.0001
Ref. [28]	7.9961	0.0005	7.9962	0.0006	7.9962	0.0005
Ref. [38]	7.9952	0.0004	7.9953	0.0003	7.9954	0.0004
Ref. [22]	7.9947	0.0005	7.9982	0.0004	7.9952	0.0004
Ref. [33]	7.9935	0.0003	7.9936	0.0002	7.9935	0.0003

**Table 14:** The means and standard deviations of the PSNRs of the cipher images obtained with the proposed approach and other tested encryption methods. The best value is shown in bold.

Methods	PSNR	
	Mean	STD
The proposed	**80.7231**	0.0000
Ref. [28]	80.7229	0.0001
Ref. [38]	80.6343	0.0002
Ref. [22]	80.4352	0.0001
Ref. [33]	80.2396	0.0002

**Table 15:** The means and standard deviations of key sensitivity NPCRs and UACIs in percentage for all tested methods on the R, G and B components of cipher images. The best values are shown in bold.

Methods		NPCR (%)			UACI (%)		
		R	G	B	R	G	B
The proposed	Mean	99.6025	**99.6110**	**99.6130**	**33.4700**	33.4835	**33.4555**
	STD	0.0152	0.0155	0.0130	0.0439	0.0589	0.0867
Ref. [28]	Mean	99.6233	99.6231	99.6232	33.4512	**33.4615**	33.4513
	STD	0.0221	0.0232	0.0124	0.1053	0.0733	0.1042
Ref. [38]	Mean	99.6002	99.6003	99.6006	33.4315	33.4326	33.4378
	STD	0.0204	0.0213	0.0193	0.1032	0.0985	0.0927
Ref. [22]	Mean	99.6203	99.6201	99.6202	33.4432	33.4428	33.4527
	STD	0.0225	0.0213	0.0204	0.0923	0.1121	0.1027
Ref. [33]	Mean	**99.6152**	99.6151	99.6153	33.4302	33.4301	33.4415
	STD	0.0227	0.0208	0.0183	0.0925	0.0834	0.1023

**Table 16:** The means and standard deviations of plain image sensitivity NPCRs and UACIs in percentage for all tested methods on the R, G and B components of cipher images. The best values are shown in bold.

Methods		NPCR (%)			UACI (%)		
		R	G	B	R	G	B
The proposed	Mean	**99.6033**	**99.6121**	**99.6107**	**33.4642**	33.4527	**33.4658**
	STD	0.0101	0.0098	0.0125	0.1017	0.1032	0.1073
Ref. [28]	Mean	99.6062	99.6053	99.6044	33.4573	**33.4582**	33.4535
	STD	0.0032	0.0011	0.0023	0.1124	0.1025	0.1327
Ref. [38]	Mean	99.5842	99.5837	99.5842	33.4435	33.4483	33.4216
	STD	0.0026	0.0034	0.0015	0.1127	0.1103	0.1114
Ref. [22]	Mean	99.5873	99.5852	99.5973	33.4592	33.4519	33.4612
	STD	0.0015	0.0026	0.0031	0.0833	0.1015	0.0954
Ref. [33]	Mean	99.5851	99.5842	99.5862	33.4532	33.4518	33.4526
	STD	0.0023	0.0021	0.0017	0.0903	0.0874	0.1217

**Table 17:** The means and standard deviations of the computation time each tested method needs to generate a cipher image. The best value is shown in bold.

Methods	Computation Time (in Seconds)	
	Mean	STD
The proposed	51.18	0.52
Ref. [28]	202.51	2.67
Ref. [38]	70.57	0.64
Ref. [22]	41.73	0.21
Ref. [33]	**35.68**	0.35

## Data Availability

The source code of the computer program developed in this study is available from the corresponding author upon request.
